# Diet-induced obesity and aging-induced upregulation of Trib3 interfere with energy homeostasis by downregulating the thermogenic capacity of BAT

**DOI:** 10.1038/s12276-024-01361-5

**Published:** 2024-12-02

**Authors:** Hyejin Yeo, Ji-Hye Lim, Ji Eom, MinJeong Kim, Hyeji Kwon, Sang-Wook Kang, Youngsup Song

**Affiliations:** 1 Department of Brain Science, Brain Korea 21 Project, Asan Medical Center, University of Ulsan College of Medicine, Seoul, Korea; 2 Department of Biochemistry and Molecular Biology, Brain Korea 21 Project, Asan Medical Center, University of Ulsan College of Medicine, Seoul, Korea

**Keywords:** Obesity, Experimental models of disease

## Abstract

Characterized by UCP1 expression and abundant mitochondria, brown adipose tissue (BAT) plays a crucial role in energy balance by converting chemical energy into heat through the cost of ATP production. In this study, it was demonstrated that Trib3 is a critical determinant of BAT-mediated energy expenditure and whole-body energy homeostasis. Under 60% high-fat diet conditions, Trib3 expression in BAT was elevated. Mice deficient in Trib3 are resistant to diet-induced obesity and exhibit improved glucose homeostasis due to enhanced BAT activity. Furthermore, brown adipocyte progenitor cells (APCs) lacking Trib3 exhibited increased proliferation and promoted brown adipocyte differentiation and mitochondrial biogenesis, contributing to the increase in the maximal thermogenic capacity of BAT in Trib3-deficient mice. Mechanistically, it was discovered that Trib3 expression is upregulated by free fatty acids at the transcriptional level and synergistically upregulated by DAG-PKC at the posttranslational level. This occurs through the modulation of COP1-mediated Trib3 protein turnover. Interestingly, the level of Trib3 expression in BAT increased with age. Trib3 knockout mice were protected from aging-related weight gain and impaired glucose homeostasis. These results suggest that Trib3 acts as an obesity- and aging-associated factor that negatively regulates BAT activity and that the loss of Trib3 may provide a beneficial approach to prevent obesity and aging-associated metabolic syndrome by increasing the thermogenic capacity of BAT.

## Trib3 deficiency boosts brown fat and metabolic health

The study investigates how the Trib3 gene influences energy balance and obesity. Researchers discovered that Trib3 knockout mice, which lack the Trib3 gene, are resistant to diet and aging-induced obesity. This study fills a gap in understanding Trib3’s role in brown adipose tissue, a type of fat that generates heat. Researchers conducted experiments on Trib3 KO mice to examine their resistance to obesity using methods like glucose tolerance tests, indirect calorimetry, and PET imaging to analyze the mice. Results showed that Trib3 KO mice had lower body weight and better glucose metabolism compared to control mice. They concluded that Trib3 KO mice have increased energy expenditure due to enhanced BAT activity. This suggests that targeting Trib3 could help treat obesity and related metabolic disorders. Future research could explore Trib3’s role in other tissues and its potential as a therapeutic target.

This summary was initially drafted using artificial intelligence, then revised and fact-checked by the author.

## Introduction

Energy homeostasis in humans is maintained through precise regulation of the balance between energy intake and expenditure. This process involves close interactions between peripheral tissues and the brain. The brain detects and integrates metabolic signals to optimize the regulation of metabolic balance, whereas peripheral tissues monitor and communicate energy storage levels to the brain^1^. Dysregulating any part of these processes can disrupt energy homeostasis and lead to metabolic disorders.

Adipose tissue is one of the most important peripheral organs for energy homeostasis. Classically, there are two distinct tissue types: white adipose tissue (WAT) and brown adipose tissue (BAT); however, a third form, namely, beige adipocytes, has recently been identified in WAT depots, which are interconvertible between WAT and BAT-like properties^2^. WAT is an endocrine organ that stores excess energy and transmits information regarding the energy state of the body via hormonal signals. However, BAT is considered closer to a catabolic organ that plays a unique role in energy-consuming adaptive thermogenesis. Morphologically, white adipocytes contain single lipid droplets that occupy most of the cell volume, whereas brown adipocytes are much smaller and contain multiple small lipid droplets. BAT derives its brown hue from the high content of hem cofactors of cytochrome oxidase in the mitochondria. Brown adipocytes are a rich source of mitochondria, which are generally larger and packed with more cristae. The presence of UCP1 allows BAT to acquire the special feature of nonshivering thermogenic activity. Similar to other cells, although brown adipocytes generate ATP from a proton gradient generated by the mitochondrial respiratory chain. However, UCP1, a gene specifically expressed in brown adipocytes and located in the inner mitochondrial membrane, acts as a channel for protons to pass through, dissipates the proton gradient, and generates heat instead of ATP^3,4^. Since UCP1-mediated uncoupling reactions do not prevent respiratory flux, entering the uncoupling futile cycle accelerates respiratory activity to meet the intracellular ATP demand, further increasing energy expenditure. Therefore, targeting BAT by increasing energy expenditure has emerged as a potential strategy for treating obesity and related metabolic disorders.

Genetic screening of *Drosophila melanogaster* revealed that the Tribbles (Trbl) gene, which is involved in cell proliferation, migration, and development^5,6^, belongs to the serine/threonine pseudokinase family and is characterized by the sharing of a kinase-like domain. The predicted Trbl kinase lacks a canonical metal-binding DFG motif and thus does not retain phosphotransferase activity or function as a kinase^7^. Despite the loss of kinase activity, Trbl, by retaining one of the key characteristics of kinases, namely, specific interactions with proteins, serves as a signaling mediator and scaffold subunit, involving a myriad of cellular and physiological processes. The first elucidated functional mechanism of Trbl in *D. melanogaster* oogenesis and gastrulation involved the modulation of Slbo and String protein turnover by directly binding and linking these proteins to the E3 ligase COP1^5,6^. Three *D. melanogaster* Trbl homologs, Tribbles 1, 2, and 3 (Trib1, 2, 3), have been identified in mammals. Although ATP binding affinity and phosphotransferase activity appear to differ slightly, they all conserve a central pseudokinase domain and exhibit both shared and unique roles through direct interaction with various proteins, including cdc25, C/EBP, 12-LOX, MEK-1, MKK4, MKK7, and ATF4^5,6,8–11^. While the diverse functions of Tribbles have been revealed, the discovery of Trib3 as an AKT binder through yeast two-hybrid screening has highlighted the metabolic roles of the Tribbles family. Fasting induces Trib3 in the liver, which directly blocks the phosphorylation of AKT and disrupts insulin-dependent suppression of hepatic glucose production. Consequently, mice with hepatic-specific Trib3 overexpression are resistant to insulin signaling, leading to type 2 diabetes^12^. Similarly, introducing Trib2 specifically into the liver of mice also leads to insulin resistance, and systemic and liver-specific deficiency of Trib2 protects mice against diet-induced obesity with improved insulin sensitivity and glucose homeostasis^13^. On the other hand, hepatocyte-specific Trib1 knockout mice exhibit hyperlipidemia, particularly those with increased total cholesterol levels^14^, and systemic Trib1 knockout mice exhibit obesity with impaired BAT-mediated thermogenesis^15^. Although multiple gain-of-function and loss-of-function studies have further elucidated the metabolic role of Trib3 in WAT and skeletal muscle^15–20^, in addition to one in vitro studies, the function of Trib3 in brown adipose tissues has rarely been examined. Here, using Trib3 KO mice, the associations between Trib3 and overnutrition, aging-related obesity, and its role in energy expenditure in brown adipocytes are investigated.

## Materials and methods

### Animals

All animal experiments were performed following an approved protocol (approval number: 2019-01-112) from the Institutional Animal Care and Use Committee of Asan Life Sciences Institute, Asan Medical Center, Seoul, South Korea. Trib3 KO mice, in which the 2.5 kb DNA fragment Trib3 gene was replaced with a neomycin resistance gene cassette^21^, were backcrossed into C57BL6/J mice (Jackson Laboratory (Stock #000664)) over a minimum of eight generations. All the mice used in this study were housed in a temperature-controlled (22–24 °C) pathogen-free facility under a 12 h light‒dark cycle with free access to water and a normal chow diet (Purina Rodent Chow, Seoul, Korea). For diet-induced obesity studies, 6–8-week-old control and Trib3 KO mice fed a normal chow diet were transferred to a 60% high-fat diet (D12942, Research Diets, New Brunswick, NJ, USA). Body weight and food consumption were monitored weekly. Magnetic resonance imaging (MRI) or dual energy X-ray absorptiometry (DEXA) scans for fat and lean mass analysis were performed via an Echo MRI-100 (Echo Medical Systems LLC, Houston, TX, USA) or iNSiGHT VET DXA (Intosia, Daegu, Korea) according to the manufacturer’s instructions.

### Glucose, insulin tolerance test (GTT, ITT)

For the GTT experiments, 16-h fasted male mice were intraperitoneally (IP) injected with glucose (1.5–2 g [glucose]/kg [mouse weight], depending on the weight of the mice). The tail vein blood glucose level was measured every 0.5 h via an Accu Check Performa glucometer (Roche, Basel, Switzerland). For the ITT, 1–1.2 U/kg insulin (Humulin, Lilly) was introduced IP into male mice that had been fasted for 4–5 h, and glucose levels were measured in the same way as those in the GTT.

### Glucose uptake assay

The glucose uptake assay was conducted as described previously^17^, according to the manufacturer’s instructions (Abcam, UK). Briefly, soleus muscle strips dissected from the hind legs of control and Trib3 KO mice were rinsed with PBS and incubated twice in Krebs-Ringer-Phosphate-HEPES buffer (KRPH) containing 2% BSA in a 5% CO_2_ incubator. After a 1-h incubation, soleus muscle strips were transferred to KRPH buffer containing 2% BSA for 40 min. Next, 10 mM 2-deoxy-D-glucose (2-DG) with or without insulin was added. After being washed twice with KRPH with 2% BSA, the tissues were lysed with extraction buffer provided by the kit, and 2-DG uptake was measured at an optical density of 412 nm (Biotek, Winooski, VT, USA).

### Plasma analysis

Circulating insulin levels were assessed via ELISA (Alpco, Salem, NH, USA) according to the manufacturer’s instructions.

### Histology

Isolated tissues from the control and Trib3 KO mice were immediately fixed with 4% paraformaldehyde for more than 24 h and embedded in paraffin. Paraffin-embedded sections were prepared at a thickness of 5 μm and subjected to hematoxylin and eosin (H&E) staining.

### Indirect calorimetry

Metabolic cage studies were conducted as previously described^22^. Beforehand, control and Trib3 KO mice were individually housed for at least 2 days for acclimation. The O_2_ consumption, CO_2_ production, locomotor activity, and food intake of the individually housed mice were monitored by an indirect calorimeter (Columbus Instruments, Columbus, OH, USA or TSE systems, Bad Homburg, Germany), and the data was analyzed 24 h after initiating the study. For β3-adrenergic receptor stimulation experiments, 100 μg/g CL316,243 (Sigma‒Aldrich) was injected IP, and metabolic cage studies were continued as described above. Heat (Kcal/h/kg), VO_2_ (ml/h/kg), and VCO_2_ (ml/h/kg) were calculated on the basis of lean body mass. Additionally, the metabolic rate was analyzed via regression-based analysis of covariance (ANCOVA), with lean body mass as a covariate^23^.

### PET imaging and analysis

Control and Trib3 KO mice fed a 60% HFD for 12 weeks were fasted overnight and subjected to 1 h of cold exposure. A total of 6.5 ± 1.0 MBq of 0.2 mL of FDG was administered to the mice via the tail vein, and PET imaging was performed via a NanoScan® PET/MRI (1 T, Mediso, Hungary) under anesthesia (1.5% isoflurane in 100% O_2_ gas). MR whole-body imaging revealed T1-weighted images with a gradient-echo (GRE) 3D sequence (TR = 25 ms, TE_eff_ = 3, FOV = 64 mm, matrix= 128 × 128), which were acquired during the FDG uptake period. Ten minutes of static PET images were acquired at 1–5 coincident points in a single field of view with the MRI range. PET images were reconstructed by Tera-Tomo 3D in full detector mode, with all corrections, high regularization, and eight iterations. Three-dimensional volume of interest (VOI) analysis of the reconstructed images was performed via the InterView Fusion software package (Mediso, Hungary), and standard uptake value (SUV) analysis was applied. VOIs fixed with a diameter of 1 mm were drawn at the BAT site. The SUV of each VOI site was calculated by the following formula: SUVmean = (tissue VOI with the unit of Bq/cc × body weight [g]) divided by injected radioactivity. %ID/g= (SUV/body weight [g] × 100%).

### Tissue cell culture

The HEK293T and pre-BAT H1B1 cell lines were cultured in Dulbecco’s modified Eagle’s medium (DMEM, Welgene, Gyeongsan, R. of Korea) supplemented with 10% fetal bovine serum (FBS, Corning, Corning Life Sciences, NY, USA) and 1% penicillin/streptomycin (PS, Corning). For free fatty acid (FFA) treatment, H1B1 cells were cultured to 80% confluence. The culture medium was then replaced with DMEM containing 0.2 mM FFAs (oleic acids) conjugated to 2% bovine serum albumin (BSA). For 12-O-tetradecanoylphorbol-13-acetate (TPA) treatment, H1B1 cells at 80% confluence were treated with 100 nM TPA dissolved in dimethyl sulfoxide. Similarly, 200 nM rapamycin, 10 μM LY294002, 10 μM MG132, 5 μM thapsigargin, or 100 μg/ml cyclohexamide was applied to H1B1 cells as indicated in the figures. To culture bSVF, BAT from control and Trib3 KO mice was isolated, minced, and incubated in 1× Krebs Ringer bicarbonate HEPES (KRBH: 3 mM HEPES, 12 mM NaCl, 0.4 mM KH_2_PO_4_, 0.1 mM MgSO_4_∙7H_2_O, 0.1 mM CaCl_2_, and 1 mM NaHCO_3_) buffer containing collagenase (Gibco, Grand Island, NY, USA) at 37 °C at 200 rpm in a shaking incubator. The supernatant was removed by centrifugation 1 h after incubation. The pellet fraction was suspended in DMEM and filtered through a 70 μm nylon cell strainer (SPL Life Sciences, Pocheon, R. of Korea). The filtered cells were washed three times with DMEM and cultured in DMEM containing 10% FBS with 1% PS.

### Adipocyte differentiation

bAPCs (bSVFs) were plated at a density of 2.5 × 10^5^ cells/well in a 24-well plate. Two days after the clonal expansion of bAPCs, induction media (DMEM containing 10% FBS, 20 nM insulin (Eli Lilly), 1 nM T3 (Sigma‒Aldrich), 0.125 mM indomethacin (Sigma‒Aldrich), 5 μM dexamethasone (ENZO Life Science), 0.5 μM rosiglitazone (Cayman Chemical Company), and 0.5 mM IBMX (Tocris Bioscience)) was added for 2 days. Then, the media was changed to differentiation media (DMEM containing 10% FBS, 20 nM insulin, and 1 nM T3), and the media was changed to fresh differentiation media every other day. For Oil Red O staining, differentiated bAPCs were washed with PBS and fixed with 4% paraformaldehyde for 1 h. Fixed cells were washed with PBS, ddH_2_O and 60% isopropanol were then applied for 5 min. Fat staining was visualized with Oil Red O staining (Sigma‒Aldrich) for approximately 1 h, and pictures were taken with a phase contrast microscope (TS100, Nikon Instech, Tokyo, Japan) with a digital camera as described previously (DSF12, Nikon Instech, Tokyo, Japan).

### Ubiquitination assay

The ubiquitination assay was conducted as previously described^24^. Briefly, plasmid DNA encoding FL-Trib3, HA-COP1, HA-DET1, and 6XHis-tagged ubiquitin was transfected into HEK-293T cells as indicated in the figures. Protein samples were prepared with lysis buffer containing 1% SDS in 100 mM Tris-Cl (pH 7.4) and diluted with HEPES buffer, and total ubiquitinated protein was precipitated with cobalt-coated Talon beads.

### Immunoblot

Protein lysates from cultured cells were prepared with ice-cold 100 mM Tris-Cl (pH 7.4) containing 1% SDS. For tissue samples, snap frozen dissected mouse tissues were ground in liquid nitrogen and lysed with RIPA buffer supplemented with a proteinase inhibitor (Roche, Basel, Switzerland) and phosphatase inhibitor. For immunoblotting analysis, the antibodies, including Trib3 (house-made), DET1, HSP90 (Santa Cruz Biotechnology, Dallas, TX, USA), UCP1, COP1 (Abcam, Cambridge, UK), Flag-M2, HA (Sigma‒Aldrich, St. Louis, MO, USA), FABP4 (AP2) (Proteintech, Rosemont, IL, USA), AKT, phosphor-AKT, ACC, phosphor-ACC, ATG7, ATG3, LC3, p62, C/EBPα, C/EBPβ, PPARγ, SDHA, PDHA, COXII, ERK, phosphor-ERK, and phosphor-PKC substrates (Cell Signaling Technology, Danvers, MA, USA), were used.

### mRNA analysis

Total RNA from cultured cells and ground tissues (described above) was extracted using an RNA Mini Kit (Favorgen, Ping-Tung, Taiwan) according to the manufacturer’s instructions. Approximately 200 ng of total RNA was used to synthesize first-strand cDNA with a random hexamer and subjected to real-time quantitative reverse transcriptase PCR (qRT‒PCR) (Toyobo, Osaka, Japan) according to the manufacturer’s instructions. The following primers were used for amplification and quantification of mRNA expression levels.

**Table:** 

Primer	Sequence (5′ → 3′)
Mouse L32-F	5′ TCTGGTGAAGCCCAAGATCG 3′
Mouse L32-R	5′ CCTCTGGGTTTCCGCCAGTT 3′
Mouse c/EBPβ-F	5′ ACTTCAGCCCCTACCTGGAG 3’
Mouse c/EBPβ-R	5′ AGAGGTVGGAGAGGAACTCG 3′
Mouse c/EBPα-F	5′ TGGACAAGAACAGCAACGAG 3′
Mouse c/EBPα-R	5′ TCACTGGTCAACTCCAGCAC 3′
Mouse PPARγ-F	5′ ATGGAAGACCACTCGCATTC 3′
Mouse PPARγ-R	5′ AACATTGGGTCAGCTCTTG 3′
Mouse ADRB3-F	5′ ACAGGAATGCCACTCCAATC 3′
Mouse ADRB3-R	5′ GGGGAAGGTAGAAGGAGACG 3′
Mouse Myf5-F	5′ CCACCAACCCTAACCAGAGA 3′
Mouse Myf5-R	5′ CTCCACCTGTTCCCTCA 3′
Mouse PRDM16-F	5′ AGGGCAAGAACCATTACACG 3′
Mouse PRDM16-R	5′ GGAGGGTTTTGTCTTGTCCA 3′
Mouse UCP1-F	5′ GGATTGGCCTCTACGACTCA 3′
Mouse UCP1-R	5′ TGCCACACCTCCAGTCATTA 3′
Mouse PGC1α-F	5′ TTGCCCAGATCTTCCTGAAC 3′
Mouse PGC1α-R	5′ TTGGTCGCTACACCACTTCA 3′
Mouse SDHA-F	5′ ACCCAGACCTGGTGGAGACC 3′
Mouse SDHA-R	5′ GGATGGGCTTGGAGTAATCA 3′
Mouse VDAC1-F	5′ TTCGTCATTCTCGCCGAACA 3′
Mouse VDAC1-R	5′ CCAACCCTCATAGCCAAGCA 3′
Mouse COX2-F	5′ ACCTGGTGAACTACGACTGCT 3′
Mouse COX2-R	5′ CCTAGGGAGGGGACTGCTCA 3′
Mouse ND2-F	5′ GCCTGGAATTCAGCCTACTAGC 3′
Mouse ND2-R	5′ GGCTGTTGCTTGTGTGACGA 3′
Mouse ND5-F	5′ AGCATTCGGAAGCATCTTTG 3′
Mouse ND5-R	5′ TTGTGAGGACTGGAATGCTG 3′
Mouse ATP6-F	5′ GGCACCTTCACCAAAATCAC 3′
Mouse ATP6-R	5′ CGGTTGTTGATTAGGACGTTT 3′
Mouse Cytochrome B-F	5′ TTTTATCTGCATCTGAGTTTAATCCTGT 3′
Mouse Cytochrome B-R	5′ CCACTTCATCTTACCATTTATTATCGC 3′
Mouse TRIB3-F	5′ GGAACCTTCAGAGCGACTTG 3′
Mouse TRIB3-R	5′ TCTCCCTTCGGTCAGGCTGT 3′
Mouse F4/80-F	5′ AGTACGATGTGGGGCTTTTG 3′
Mouse F4/80-R	5′ CATCCCCCATCTGTACATCC 3′

### Statistics

The data is presented as the means ± SEMs. Statistical significance was assessed via ANCOVA or an unpaired Student’s *t* test, as appropriate. *P* values are indicated as follows: *P < 0.05, **P < 0.01, ***P < 0.001, with values less than 0.05 considered statistically significant.

## Results

### Trib3 KO mice are resistant to diet-induced obesity

**Fig. 1:**
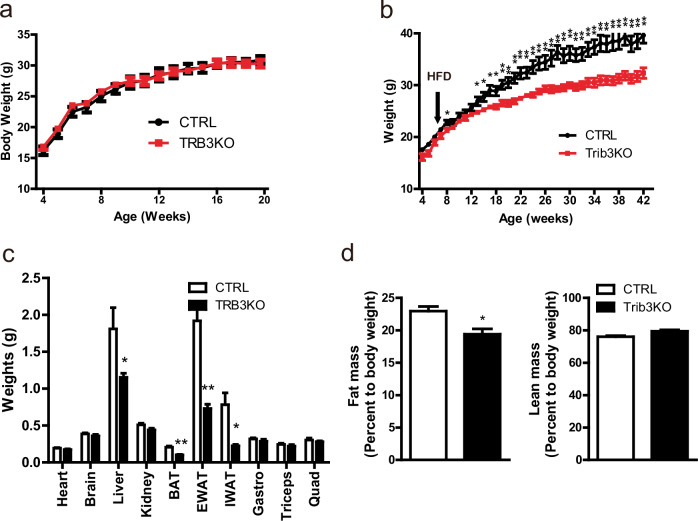
Trib3 KO mice are resistant to diet-induced obesity. Relative weight gain in control (wild-type) and Trib3 KO mice fed (**a**) normal chow diet or (**b**) 60% high-fat diet (*n* = 5 per group). **c** Weights of tissues from 42-week-old control and Trib3 KO mice fed a 60% HFD (*n* = 4–5 per group). **d** DEXA analysis showing the relative fat and lean mass/body weight ratios of 42-week-old control and Trib3 KO mice fed a 60% HFD (*n* = 4 per group).

The body weights and weights of the major metabolism-related tissues of Trib3 KO mice fed a normal chow diet were similar to those of the control mice (Fig. 1a and Supplementary Fig. 1a). Glucose and insulin tolerance tests suggested that Trib3 KO mice had systemic insulin sensitivity comparable to that of control mice (Supplementary Fig. 1b, c). Switching the Trib3 KO mice to a 60% high-fat diet (HFD) stimulated body weight gain; however, the Trib3 KO mice were resistant to 60% HFD-induced body weight gain, and the weight gain of the Trib3 KO mice was 33% lower than that of the control mice (Fig. 1b). The liver and adipose tissues of the Trib3 KO mice also weighed significantly less than those of the control mice did (Fig. 1c). DEXA analysis revealed that fat mass and lean body mass were lower in the 60% HFD-fed Trib3 KO mice than in the 60% HFD-fed control mice (Supplementary Fig. 2); however, the fat mass relative to body weight, but not the lean body mass to body weight, of the Trib3 KO mice was 20% lower than that of the control mice (Fig. 1d).

### Improved glucose metabolism in Trib3 KO mice with DIO

**Fig. 2:**
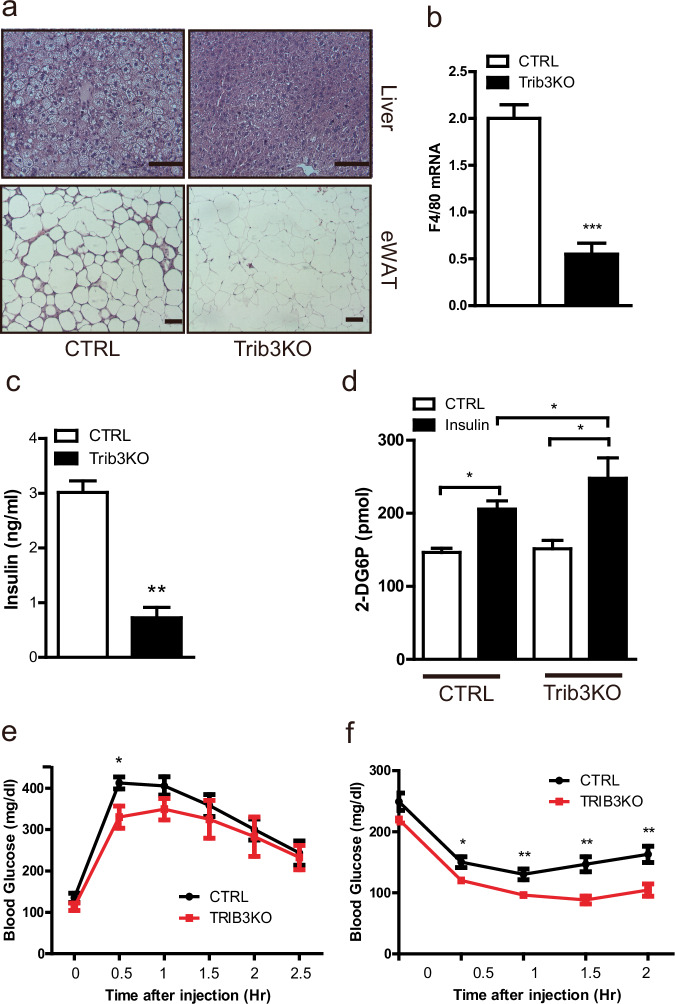
Improved glucose homeostasis in Trib3 KO mice under diet-induced obesity conditions. **a** Hematoxylin‒eosin staining of hepatic (top) and epididymal white adipose tissue (bottom) sections from control and Trib3 KO mice. **b** qRT‒PCR analysis of the mRNA levels of the macrophage-specific gene F4/80 in WAT from 60% HFD-fed control and Trib3-KO mice (*n* = 4 per group). **c** Circulating insulin levels in control and Trib3-KO mice (*n* = 4 per group). **d** Ex vivo glucose uptake assay of soleus muscles from control and Trib3 KO mice. **e** Glucose tolerance testing was conducted at 20 weeks of age, and **f** insulin tolerance testing was conducted at 24 weeks of age (*n* = 7 per group).

The hepatic steatosis and adipocyte hypertrophy observed in the control mice fed a 60% HFD were alleviated in the Trib3 KO mice (Fig. 2a). In adipose tissue, macrophage infiltration, as assessed by histology, and F4/80 expression were significantly lower in Trib3-KO mice than in control mice (Fig. 2a, b). In the context of reduced hepatic steatosis and adipose tissue macrophage infiltration, Trib3 KO mice maintained lower circulating insulin levels and exhibited increased insulin-dependent skeletal muscle glucose uptake than did control mice (Fig. 2c, d). Furthermore, consistent with previous observations of Trib3’s role in regulating ACC protein turnover and AKT/AMPK activity^12,18,19^, the phosphorylated form of AKT was upregulated in the liver, and total ACC protein levels as well as AKT and AMPK activity, as assessed by AKT and ACC (direct target of AMPK) phosphorylation levels, were upregulated in WAT (Supplementary Fig. 3). Similarly, the systemic insulin sensitivity of Trib3 KO mice improved, as shown by insulin and glucose tolerance tests (Fig. 2e–f).

### Increased energy expenditure in Trib3 KO mice

**Fig. 3:**
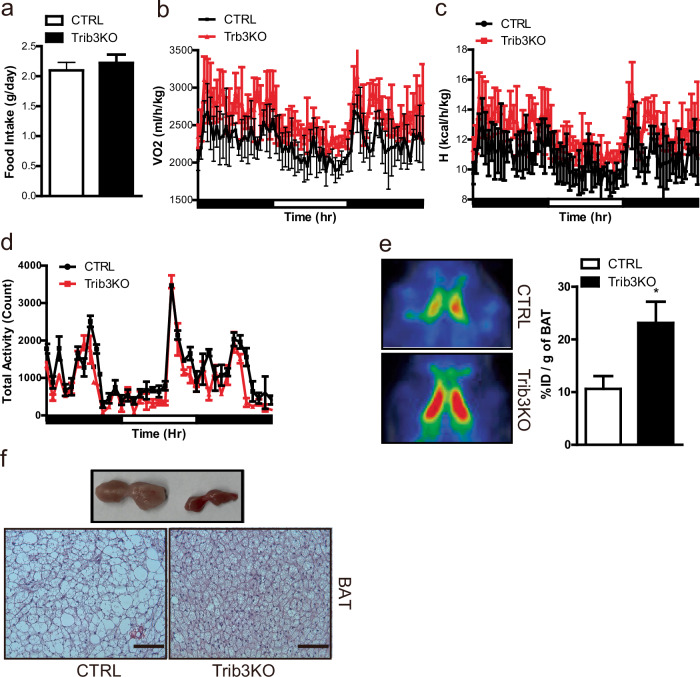
Increased energy expenditure and increased BAT activity in Trib3 KO mice. Metabolic cage analysis of (**a**) food intake, (**b**) oxygen consumption, (**c**) energy expenditure, and (**d**) physical activity in 30-week-old control and Trib3 KO mice under 60% high-fat diet conditions (*n* = 4 per group). **e** Representative image of positron emission tomography (PET) analysis of ^18^F-fluorodeoxyglucose (FDG) uptake in control and Trib3 KO mice (*n* = 4 per group). **f** Pictures of BAT depots dissected from 42-week-old control and Trib3-KO mice fed a 60% high-fat diet for 36 weeks (top) and hematoxylin‒eosin (H&E) staining of BAT sections (bottom).

The greater body weight gain in Trib3 KO mice than in control mice can be attributed to lower energy intake, increased energy expenditure, or a combination of the two. Notably, the 24-h food intake of Trib3 KO mice did not differ from that of control mice (Fig. 3a). It was subsequently considered that the reduced body weight of Trib3 KO mice could be associated with increased energy expenditure. Metabolic cage studies demonstrated that the rates of oxygen consumption were elevated in Trib3 KO mice, resulting in increased energy expenditure compared with that in control mice (Fig. 3b, c). Despite the increased energy expenditure, the physical activity of the Trib3 KO mice was almost identical to that of the controls (Fig. 3d). However, positron emission tomography (PET) analysis with ^18^F-fluorodeoxyglucose (FDG) revealed that Trib3 KO mice had a significantly greater rate of BAT glucose uptake than did control mice (Fig. 3e). The BAT subsequently became redder, and the lipid droplet size of brown adipocytes in Trib3 KO mice was smaller than that in control mice (Fig. 3f).

### Trib3 modulates brown adipocyte differentiation

**Fig. 4:**
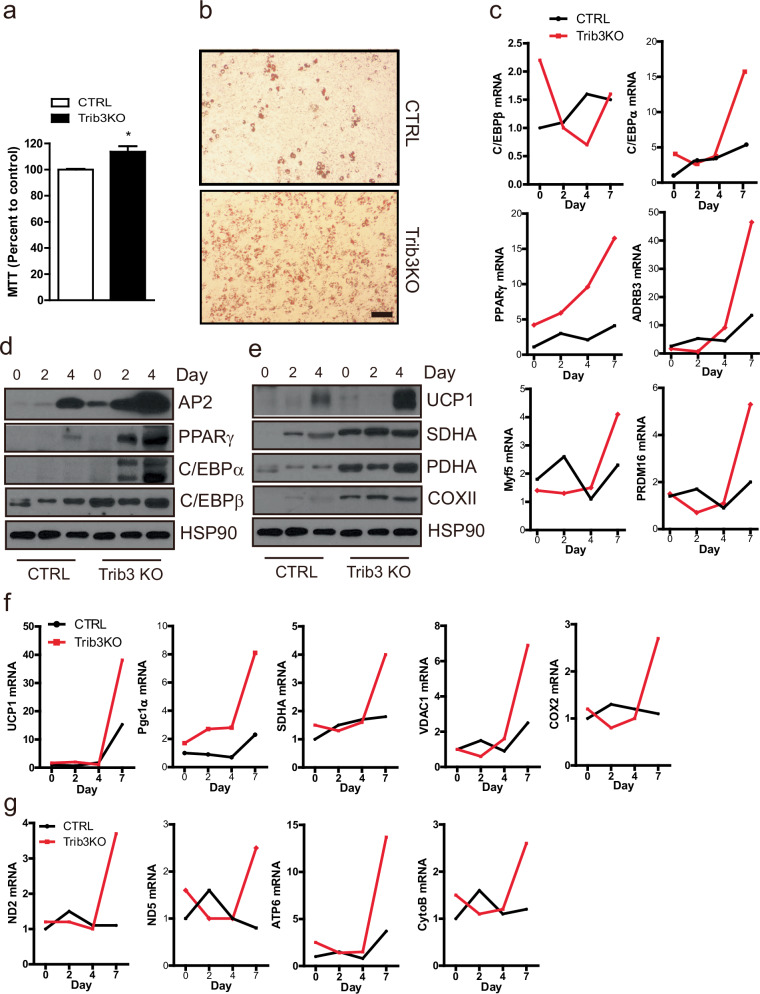
Enhanced brown adipocyte differentiation of adipocyte progenitor cells from the BAT of Trib3 KO mice. **a** Proliferation rate of brown adipose tissue-derived adipocyte progenitor cells (bAPCs; stromal vascular fraction cells) from control and Trib3 KO mice determined by MTT assay (*n* = 5–6 per group). **b** Microscopy images showing Oil Red O staining of differentiated bAPCs from control and Trib3-KO mice. **c** qRT‒PCR analysis of the mRNA expression levels of adipogenic markers during differentiation. **d** Western blot analysis of the protein expression levels of adipogenic markers during differentiation. **e** Protein levels of mitochondrial markers during the differentiation of bAPCs from control and Trib3 KO mice. **f** qRT‒PCR analysis of the mRNA expression levels of chromosome-encoded mitochondrial genes during the adipogenesis of bAPCs from control and Trib3 KO mice. **g** qRT‒PCR analysis of the mRNA expression levels of mitochondrial-genome-derived mitochondrial markers during adipogenesis in bAPCs from control and Trib3 KO mice.

To elucidate the mechanism underlying the enhanced BAT activity observed in Trib3 knockout (KO) mice, it was first examined that the signaling pathways altered by Trib3 deficiency in BAT. Analysis of signaling pathways in the BAT of mice fed a 60% HFD revealed that the phosphorylation of ACC at Ser79, a direct target of AMPK, was increased in the BAT of Trib3-KO mice compared with that of control mice (Supplementary Fig. 4a). Given that AMPK and Trib3 are well-known autophagy regulators and that autophagy plays a critical role in the regulation of BAT development and activity^17,22,25,26^, it was investigated whether autophagy is promoted in the BAT of Trib3 KO mice. Although the ATG7 protein level in the BAT of Trib3 KO mice appeared to be slightly elevated, the protein levels of ATG3, SQSTM1 (p62), and LC3, which are markers that reflect autophagy flux, were comparable to those in control mice (Supplementary Fig. 4b). Then, adipocyte progenitor cells (bAPCs) were isolated from both control and Trib3 KO mice and conducted a comparative analysis of their adipogenic capabilities. Remarkably, bAPCs from Trib3 KO mice presented 10% accelerated proliferation rates and, upon exposure to the adipogenic mixture, demonstrated a notable increase in the production of Oil Red O-positive cells compared with bAPCs from control mice (Fig. 4a, b). Similarly, the mRNA and protein levels of general adipogenic markers, such as C/EBPβ, C/EBPα, PPARγ, and β3-adrenergic receptor (ADRB3), were also greater in differentiated bAPCs from Trib3 KO mice than in those from control mice (Fig. 4c, d). Intriguingly, the expression levels of genes encoded by mitochondria, along with mitochondrial-targeted genes, as well as the key regulator of mitochondrial biogenesis Pgc1α, were also found to be elevated in differentiated bAPCs from Trib3 KO mice relative to those from controls (Fig. 4e–g).

### Increased thermogenic capacity of BAT in Trib3 KO mice

**Fig. 5:**
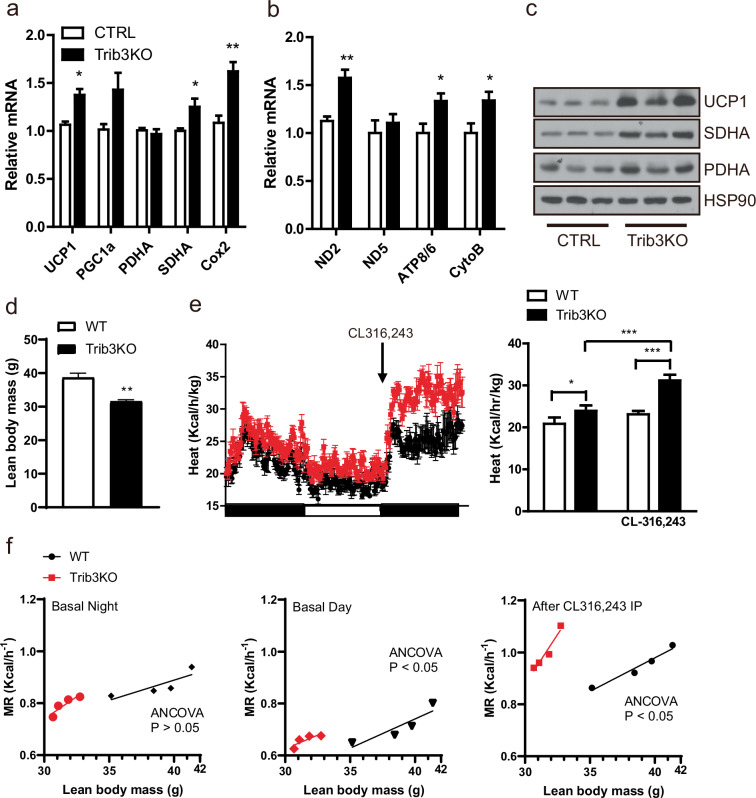
Increased thermogenic capacity of BAT in Trib3 KO mice. The mRNA levels of (**a**) chromosome-encoded mitochondrial genes and (**b**) mitochondria-derived mitochondrial genes in BAT from control and Trib3 KO mice were assessed via qRT‒PCR (*n* = 4 per group). **c** Protein levels of mitochondrial genes in BAT from control and Trib3-KO mice. Metabolic cage analysis of (**d**) lean body mass and **e** energy expenditure in control and Trib3 KO mice fed a 60% high-fat diet, with intraperitoneal administration of CL316,243, normalized to lean body mass (*n* = 4 per group). **f** Regression-based analysis-of-covariance (ANCOVA) using genotype as the fixed variable and lean body mass as the covariate.

Consistent with the in vitro results in cultured bSVFs (Fig. 4), BAT in Trib3 KO mice expressed higher levels of mitochondrial-encoded genes and chromosomal mitochondrial-targeting genes than in the control group (Fig. 5a–c). Increased mitochondrial biogenesis and UCP1 expression suggest that the increased energy expenditure in Trib3 KO mice may be due to the increased thermogenic capacity of BAT^27^. Thus metabolic cage studies, in which CL316,243, a selective ADRB3 agonist that maximizes the oxygen consumption rate was applied via the activation of the cAMP signaling pathway^28^, were conducted. In line with the previously acquired data (Fig. 3), the daytime basal rates of oxygen consumption and energy expenditure in Trib3 KO mice were elevated, and acute administration of CL316,243 increased the oxygen consumption rate and energy expenditure in both sets of mice but to a significantly greater extent in the Trib3 KO mice (Fig. 5d–f and Supplementary Fig. 5a–d). The UCP1 protein levels in the inguinal WAT of Trib3 KO mice were comparable to those in the inguinal WAT of control mice fed a 60% high-fat diet (HFD), suggesting that the contribution of beige adipocytes to the increased energy expenditure in Trib3 KO mice might be minimal compared with that in BAT (Supplementary Fig. 5e).

### A HFD upregulates Trib3 expression

**Fig. 6:**
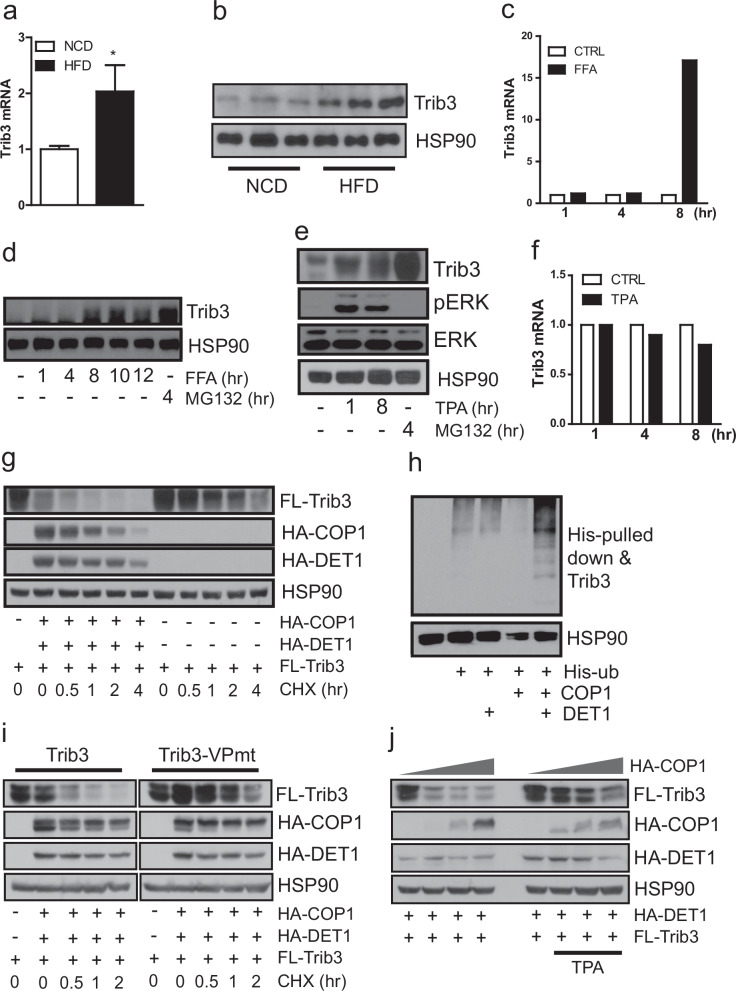
Upregulation of Trib3 expression in BAT from mice with diet-induced obesity. **a** mRNA and (**b**) protein expression levels of Trib3 in BAT from normal chow-fed and 60% high-fat-diet-fed mice. **c** mRNA and (**d**) protein expression levels of Trib3 in preadipocyte cell lines after free fatty acid (FFA) treatment. **e** Protein and **f** mRNA expression levels of Trib3 in preadipocyte cell lines after TPA treatment. **g** Protein expression levels of Trib3 in the absence or presence of COP1 and DET1. **h** Increased ubiquitination of Trib3 by COP1 and DET1 coexpression. **i** Resistance to COP1- and DET1-mediated downregulation of Trib3-VP mutant protein levels. **j** Resistance to COP1- and DET1-stimulated downregulation of Trib3 protein levels by TPA treatment.

Next, the cause of the Trib3 KO phenotype observed under HFD-fed conditions was investigated. In line with previous reports of upregulated Trib3 expression in the liver, WAT, and skeletal muscle tissue under conditions of diet-induced obesity^12,17^, it was observed that elevated Trib3 levels in the BAT of mice fed a 60% HFD compared with those in control mice (Fig. 6a, b). The accumulation of FFA, a well-known inducer of ER stress^29^, and diacylglycerol (DAG), a second messenger and a potent agonist of PKC, in peripheral tissues is a prominent cause of impaired energy metabolism in obese individuals^30,31^ (Supplementary Fig. 6a). We speculated that the increase in Trib3 expression under conditions of diet-induced obesity could be associated with FFAs, the DAG‒PKC signaling axis, or both. In line with the upregulation of Trib3 expression by ER stress^32^, FFA treatment increased Trib3 mRNA and protein levels in the pre-BAT cell Line H1B1 (Fig. 6c, d). Further, the expression of Trib3 was compared by treating H1B1 cells with TPA to determine whether Trib3 expression levels are regulated in part through the DAG‒PKC signaling axis. TPA treatment increased Trib3 protein levels without altering Trib3 mRNA levels, suggesting that the upregulation of Trib3 protein levels by PKC is a posttranscriptional process (Fig. 6e, f and Supplementary Fig. 6b). Although generally known as a scaffold protein linking the E3 ligase COP1 to its target protein^5,6,18^, we observed downregulation of the protein level of Trib3 through ubiquitination by COP1 (Fig. 6g, h). The mutant Trib3 (Trib3-VPmt), which disables binding to COP1, was resistant to COP1-stimulated degradation (Fig. 6i). Similarly, mutant COP1 (COP1-ΔRing), which cannot bind Trib3, appeared to be less efficient at degrading Trib3 (Supplementary Fig. 6c). Furthermore, TPA treatment attenuated COP1-mediated Trib3 degradation (Fig. 6j). The protein levels of COP1 and DET1 in the BAT of 60% HFD-fed mice were comparable to those in normal chow-fed mice (Supplementary Fig. 6d), suggesting that upregulated PKC activity under obese conditions inhibits COP1 and DET-induced Trib3 protein turnover.

### Trib3 is associated with age-related weight gain and insulin resistance

**Fig. 7:**
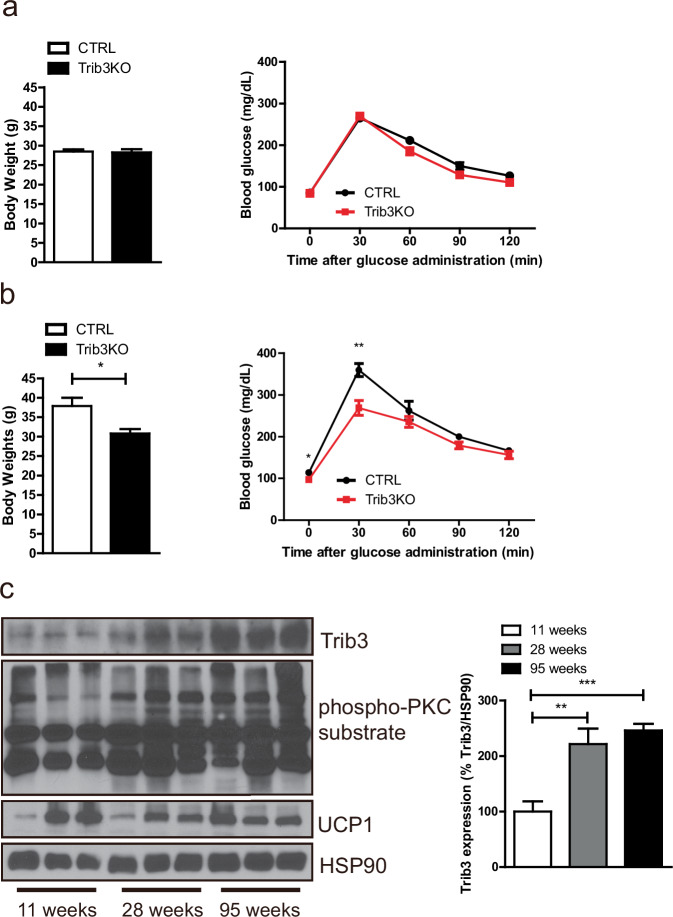
Upregulation of Trib3 protein levels in the BAT of aged mice. **a** Body weight (left) and glucose tolerance testing (right) of 18-week-old control and Trib3 KO mice. **b** Body weight (left) and glucose tolerance testing (right) of 56-week-old control and Trib3 KO mice. **c** Protein levels of Trib3 and UCP1 and PKC activity in the BAT of C57BL6/J mice at 11, 28, and 95 weeks were measured by immunoblotting (left) and quantified via densitometry (right).

During extended periods of observation, it was noted that the body weight and glucose homeostasis of Trib3 KO mice, which were initially indistinguishable from those of the control group under normal chow intake conditions, could be distinguished from those of the control group by the age of 1 year (Fig. 7a, b and Supplementary Fig. 7). After examining the correlation between aging and Trib3, it was noted that the expression level of Trib3 and PKC activity in BAT increased with aging, whereas the expression of UCP1 decreased (Fig. 7c).

## Discussion

Trib3 appears to be a highly complex protein that performs both metabolically beneficial and detrimental functions depending on the tissue. It was demonstrated that the systemic depletion of Trib3 overall is metabolically beneficial. Trib3 KO mice are resistant to diet-induced obesity, resulting in reduced adiposity and body weight and better maintenance of glucose homeostasis. The reduced body weight gain in Trib3 KO mice appears to be attributed to increased energy expenditure due to increased BAT activity. Originally believed to exist in humans only in the neonatal period, BAT has been a relatively unattractive research area. However, the identification and mapping of active human BAT in adults through a series of ^18^F-FDG-PET/CT studies in the late 2000s have accelerated research in the field. BAT is a small organ that accounts for only up to 5% of body weight but can be responsible for 75% and 50% of ingested glucose and triglyceride clearance, respectively, when fully activated^33^. In principle, BAT-mediated energy expenditure is determined by the thermogenic activity and capacity of BAT, both of which are ultimately regulated by adrenergic signals through the sympathetic nervous system. The former involves the regulation of the thermogenic activity of brown adipocytes themselves, such as triglyceride (TGA) degradation, the regulation of fatty acid oxidation, and UCP1 activity. In contrast, the latter involves the regulation of BAT cell proliferation, mitochondrial biosynthesis, and UCP1 expression. Although the possibility related to the alteration of thermogenic activity cannot be excluded, the in vitro brown adipocyte differentiation and metabolic cage data of Trib3 KO mice suggest that improved BAT activity in Trib3 KO mice was due to increased thermogenic capacity. In contrast to Trib3, Trib1 expression increases through adrenergic stimuli to promote mitochondrial respiration, indicating that Trib1 KO mice have impaired β3-adrenoreceptor-induced BAT activation, leading to obesity^34^. The reason why Trib1 and Trib3 have acquired opposite functions remains unclear; however, speculation suggests that the segregation of Trib1 and Trib3 genes evolved into positive and negative feedback regulators, respectively, to refine and maintain the homeostasis of BAT activity.

With respect to the role of Drosophila tribbles in development, mammalian Trib3 is also involved in the differentiation of several progenitor cells. In adipose tissue, the mammalian Tribbles act as antiadipogenic factors. Trib3, which is expressed abundantly in preadipocytes, is transiently downregulated during the clonal expansion phase of adipogenesis, and the overexpression of Trib2 or Trib3 suppresses the differentiation of 3T3L1 preadipocytes by directly interacting with C/EBPβ, a key component of adipogenesis^35^. In line with our findings, Jeong et al. reported that Trib3 overexpression in immortalized brown preadipocytes inhibited their differentiation into brown adipocytes in an IRS1-AKT signaling pathway-dependent manner^36^. Several studies have demonstrated that cell proliferation and differentiation are highly coupled processes with the involvement of cell cycle inhibitors in lipogenesis and in different stages of adipogenesis^37,38^. Additionally, the protein retinoblastoma (pRB) can bind to the PGC1α promoter and repress its transcription, and whereas downregulation of pRB promotes differentiation toward brown adipocytes^39^. In this context, because bSVFs from Trib3 KO mice proliferate faster than those from control mice do, it may be possible that Trib3 deletion-induced increases in proliferation rates could be additional mechanisms of enhanced differentiation of Trib3-deficient bSVFs into brown adipocytes. Determining a potential correlation between increased cell proliferation in Trib3 bSVFs and CDC25, as in the Drosophila system, or with an entirely different mechanism would be highly interesting^40^. Further research could also identify whether the enhanced brown adipogenesis of bSVFs from Trib3 KO mice is associated with the repression of RB expression or activity.

Accumulating evidence indicates that Trib3 is a stress-sensitive gene whose transcripts are regulated in response to various stress signals, such as ER stress, oxidative stress, and hypoxia^32^. The expression of Trib3 in the metabolic state is considerably more dynamic and is upregulated under energy-deficient conditions, such as essential amino acid and glucose deficiency, and under overnutrition conditions, such as excess FFAs and glucose^41,42^. Accordingly, Trib3 expression is elevated in major metabolism-related tissues of humans and rodents under fasting conditions and nutrient-excess conditions such as obesity or diabetes^12,16,17,43–46^. The results showing increased Trib3 expression in BAT in response to HFD and FFA treatment are consistent with the above literature, suggesting that the mechanisms of increased Trib3 expression in an overloaded energy state are also conserved in BAT.

In addition to the transcriptional regulation of Trib3 expression, Trib3 levels are regulated at the posttranslational level^47^. The Trib3 protein turnover rate is rapid, and despite extensive levels of Trib3 transcription, detection of the Trib3 protein by, for example, western blotting was often quite challenging until the proteasome blocker MG132 was provided. While the E3 ligase Seven in absentia homolog 1 has been shown to directly interact with Trib3 and stimulate the ubiquitination and degradation of Trib3^48^, the present study revealed that COP1 not only uses Trib3 as an adapter for linking to substrate proteins but also regulates the stability of the Trib3 protein itself. Indeed, it was observed that the protein levels of Trib3 were downregulated when COP1 and DET1 were cointroduced, but the Trib3-VP mutant, which is unable to interact with COP1, was resistant to COP1-DET-mediated degradation. COP1 activity has been shown to be negatively regulated by ERK1/2 and PKC^49,50^, which is consistent with the data indicating that COP1-DET-mediated Trib3 degradation is attenuated by TPA treatment. DAG is an endogenous agonist of both novel PKC (nPKC) and classical PKC (cPKC). However, because full activation of cPKC requires additional Ca^2+^ physiologically, the acquired Trib3 protein stability under DIO was more likely due to activation of nPKC than to activation of a cPKC family member. This speculation is also consistent with the increased activity of nPKC, particularly PKC-δ and PKC-ε, rather than cPKC in DIO and its association with insulin resistance^30,51,52^.

Importantly, it was observed that Trib3 expression levels increased with age. Trib3 KO mice, which initially showed no difference in body weight or glucose tolerance, gained less weight and exhibited lower body weight and improved glucose tolerance than control mice did by 1 year of age. Notably, the expression and activity of key ER chaperones^53,54^ and the rate of autophagy formation and maturation decrease with age^55^, which leads to the accumulation of unfolded insoluble proteins and subsequent ER stress^56^. Additionally, the accumulation of DAG in multiple tissues, including adipose tissue, in combination with aging^57^ is likely to synergistically contribute to the age-dependent upregulation of Trib3.

In conclusion, the present study demonstrated that the expression of Trib3 is regulated at the transcriptional and posttranslational levels and that DIO and aging-induced increases in FFA and DAG accumulation are likely responsible for the upregulation of Trib3 expression. While age-dependent decline in thermogenic activity and BAT capacity are well documented, little is known about their molecular mechanisms^58,59^. The data suggest that overnutrition and aging-dependent increases in Trib3 expression are at least partly responsible for the decline in the thermogenic activity of BAT and subsequent obesity and insulin resistance. Recently, a small compound that reduces lipogenic gene expression by stimulating Trib1 expression was discovered^60^. The development of drugs that suppress the expression of Trib3 through the transcriptional regulation or activation of PKC, which specifically regulates COP1 activity, could be a potential strategy to prevent and treat diet- and aging-associated obesity and type 2 diabetes. Considering that Trib3 expression is likely ubiquitous, one limitation of the current study is the use of a systemic KO model. Although our data indicate that BAT is the major contributor to the increased energy expenditure observed in Trib3 KO mice, the possibility of contributions from other tissues cannot be excluded. No other study has analyzed the functional role of Trib3 in a tissue- and time-specific manner. Future studies should assess energy expenditure at thermoneutral temperatures and employ a conditional KO approach to provide a more precise and detailed analysis of Trib3 functions in metabolism.

## Supplementary information

Supplementary Information
