# Supplementary material for: Diet-induced obesity and aging-induced upregulation of Trib3 interfere with energy homeostasis by downregulating the thermogenic capacity of BAT
Source: Exp Mol Med. 2024 Dec 2;56(12):2690–702. doi: 10.1038/s12276-024-01361-5 (PMC11671538; doi:10.1038/s12276-024-01361-5)
Supplement: Supplementary file 1 — Supplementary Information [file 12276_2024_1361_MOESM1_ESM.pdf]

Supplementary information

Supplementary Figures

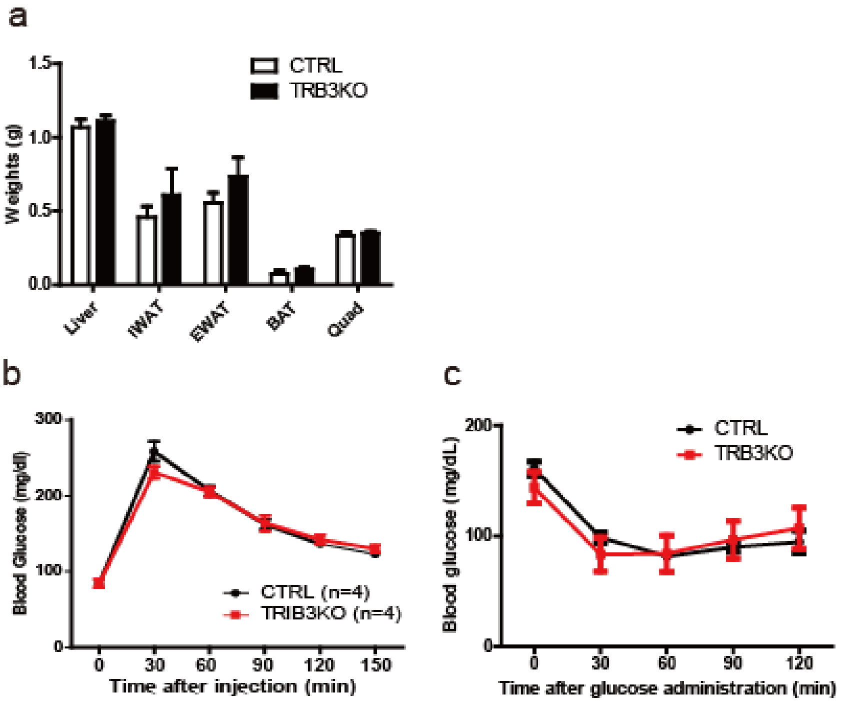

Supplementary Fig. 1. **a** The weight of tissues from 16-week-old control and Trib3 KO mice fed normal chow diets (n=4 per group). **b** Glucose and **c** insulin tolerance testing of control and Trib3 KO mice (n=4 per group).

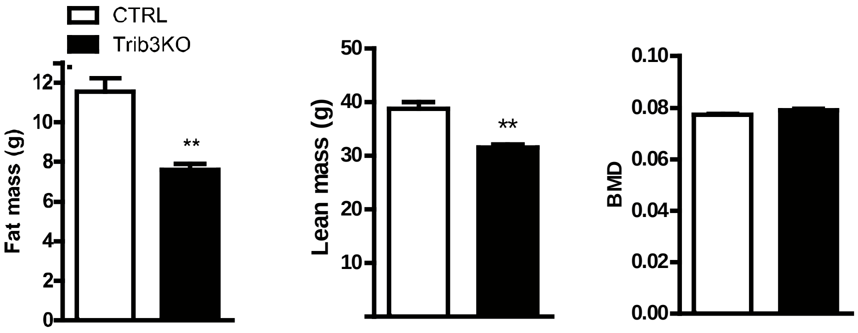

Supplementary Fig. 2. DEXA analysis showing fat and lean mass and bone mineral density for 30-week-old control and Trib3 KO mice on a 60% HFD (n=4 per group).

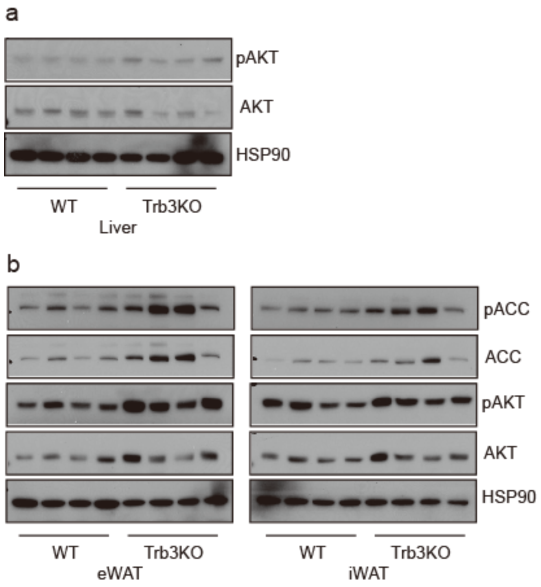

Supplementary Fig. 3 Western blot analysis of protein expression levels of total and phosphorylated forms of AKT and ACC to assess AKT and AMPK activity, respectively, in **a** liver, **b** epididymal WAT (eWAT), and c inguinal WAT (iWAT) of control and Trib3 KO mice fed a 60% high-fat diet (HFD)

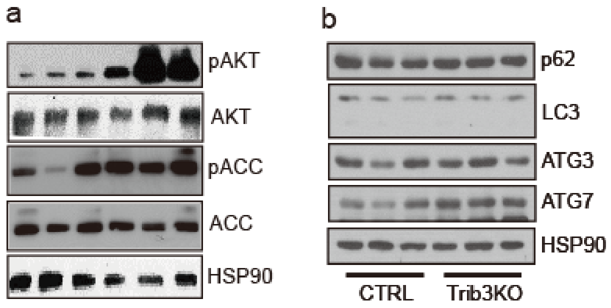

Supplementary Fig. 4 Western blot analysis of protein expression levels of **a** total and phosphorylated forms of AKT, ACC and **b** autophagy-related proteins in BAT of control and Trib3 KO mice fed a 60% high-fat diet (HFD)

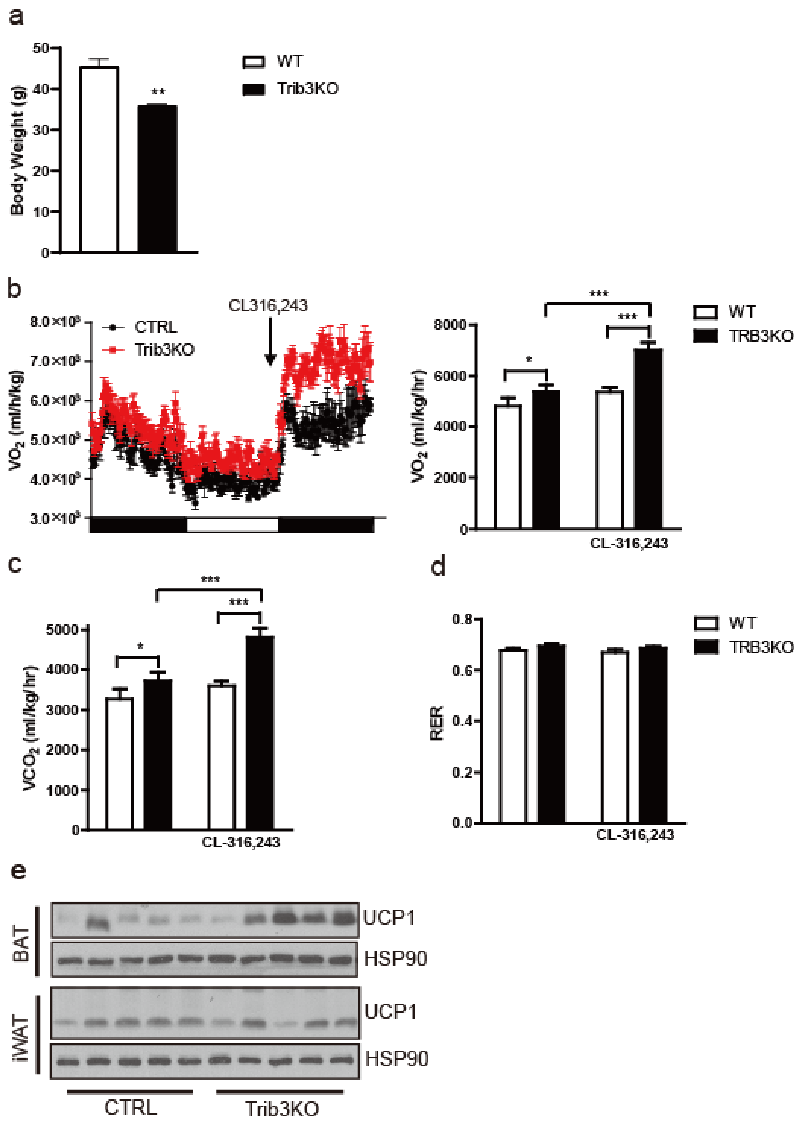

Supplementary Fig. 5. Metabolic cage analysis of **a** total body weight **b** oxygen consumption, **c** carbon dioxide production, **d** RER before and after intraperitoneal administration of CL316,243 in 30-week-old control and Trib3 KO mice under 60% high-fat diet conditions (n=4 per group). **e** protein levels of UCP1 in BAT and inguinal WAT of control and Trib3 KO mice fed 60% HFD.

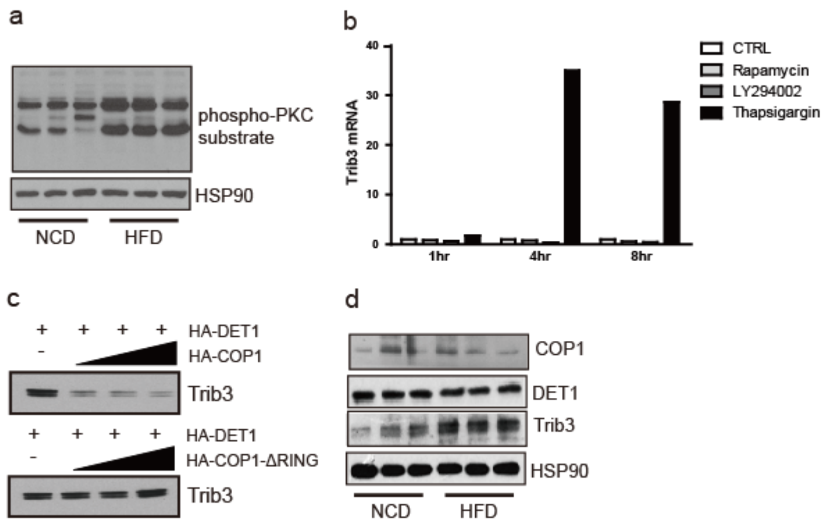

Supplementary Fig. 6. **a** PKC activity was assessed by immunoblot with phopho-PKC substrate antibody in the BAT of normal chow-fed and 60% high-fat-diet-fed C57BL6/J mice. **b** mRNA expression levels of Trib3 in preadipocyte cell lines treated with rapamycin (mTOR inhibitor), LY294002 (PI3K inhibitor), and thapsigargin (SERCA inhibitor that stimulates ER stress).**c** Protein levels of Trib3 following co-expression of wild-type COP1 or Trib3 interaction-defective COP1 mutant (COP1-ΔRing) with DET1. **d** Protein levels of COP1, DET1, and Trib3 in BAT of normal chow-fed or 60% HFD-fed C57BL6/J mice at 16 weeks old

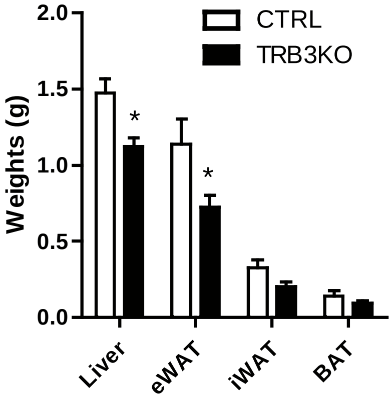

Supplementary Fig. 7. A. The weight of tissues from 1 year old control and Trib3 KO mice fed normal chow diets (n=5 per group).
